# Diagnostic utility of immunohistochemistry in detection of *NPM1* mutations in acute myeloid leukemia with a patchy distribution

**DOI:** 10.1002/jha2.866

**Published:** 2024-02-19

**Authors:** Qing Wei, Sa A. Wang, Sanam Loghavi, Hong Fang, L. Jeffrey Medeiros, Wei Wang

**Affiliations:** ^1^ Department of Hematopathology University of Texas MD Anderson Cancer Center Houston Texas USA

**Keywords:** acute myeloid leukemia, immunohistochemistry, NPM1

## Abstract

Nucleophosmin 1 (*NPM1*) mutations occur in approximately one‐third cases of adult de novo acute myeloid leukemia (AML). Identification of *NPM1* mutations is important for classification, risk stratification, tailored therapy, and monitoring minimal residual disease. Mutational analysis is widely used for detecting *NPM1* mutations. Immunochemistry assessing abnormal cytoplasmic localization of NPM1 protein has been used as a surrogate marker for *NPM1* mutations. We present a case of AML with mutated NPM1 that was missed by sequencing analysis but detected by immunohistochemistry. This case highlights the value of immunohistochemistry in identifying *NPM1* mutations in a subset of AML cases.

## 1 INTRODUCTION

Nucleophosmin 1 (*NPM1*) is mutated in approximately 30% of adult de novo acute myeloid leukemia (AML) cases [1]. AML with *NPM1* mutation is recognized as a distinct entity in the 2022 World Health Organization of hematopoietic neoplasms [2] and International Consensus Classification of Hematopathology [3]. Identification of *NPM1* mutation in AML is important for diagnosis, prognostic stratification, clinical management, and monitoring minimal residual disease [4, 5]. Patients with *NPM1* mutations without FLT3‐negative internal tandem duplication (ITD) have a more favorable prognosis than those with the FLT3‐ITD and are usually not offered allogeneic stem cell transplant [6].

Over 95% of *NPM1* mutations occur at exon 12 [4, 7, 8]. Rare mutations involve other exons (exons 5, 9, and 11) or *NPM1* translocations [9, 10, 11, 12]. These changes lead to loss of the nucleolar localization signal and formation of a nuclear export signal motif resulting in aberrant cytoplasmic localization of NPM1 protein [4, 7–12]. Immunohistochemistry (IHC) to detect cytoplasmic NPM1 can be used as a surrogate for *NPM1* mutations involving exon 12 or other uncommon sites as well as *NPM1* translocations.

Here, we report a case of AML in which cytoplasmic NPM1 was detected by IHC, but mutation was initially not detected by next‐generation sequencing (NGS) analysis. With knowledge of the IHC result, manual review of the NGS results revealed a canonical mutation of *NPM1* with very low allele frequency(<2%), below the validated reportable threshold of 5% in our laboratory. Morphologic evaluation of the bone marrow specimen showed a patchy distribution of blasts, leading to a false‐negative *NPM1* mutation by NGS. This case illustrates the value of NPM1 IHC in diagnostic workup of AML.

## 2 CASE REPORT

**FIGURE 1:**
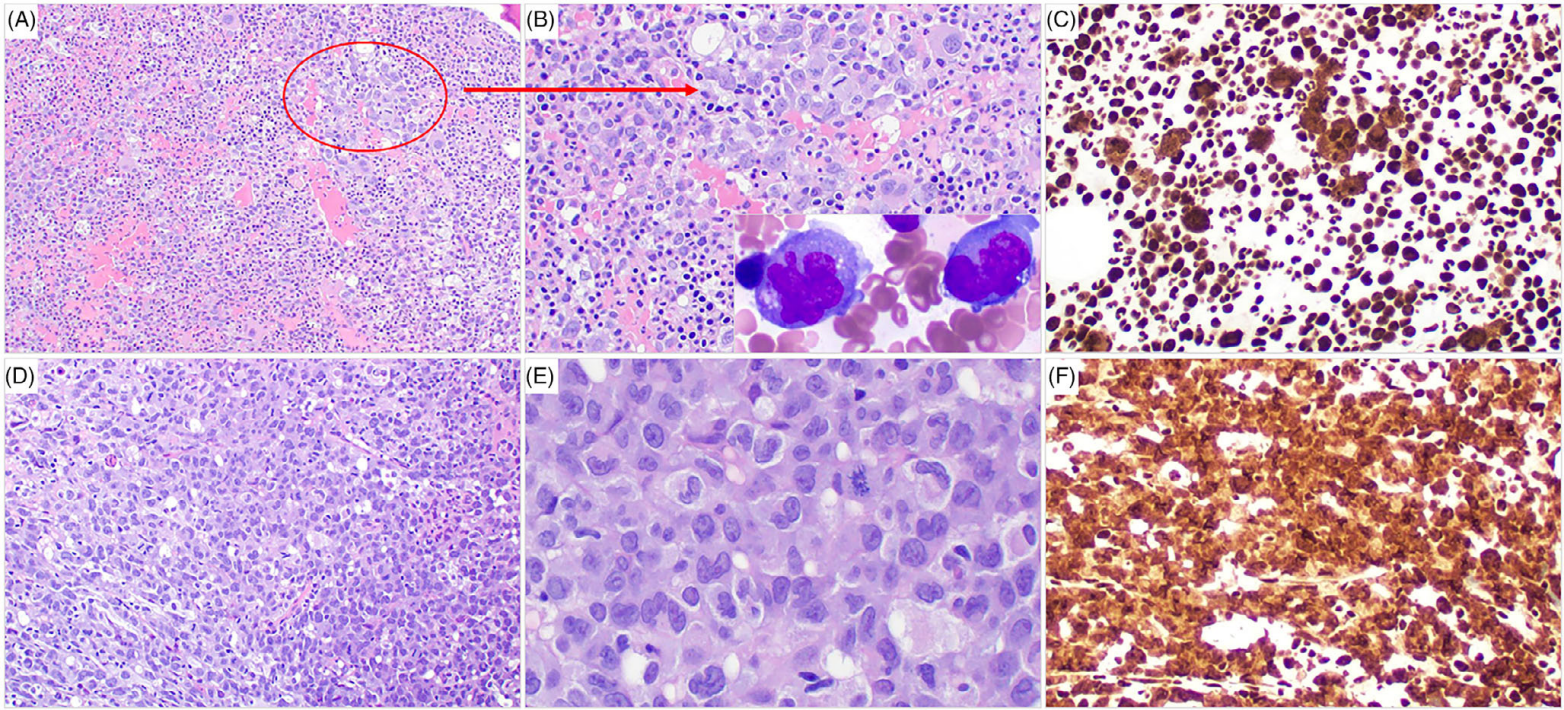
Blasts in this case of nucleophosmin 1 (*NPM1*) mutated acute myeloid leukemia (AML) were present in a patchy distribution and immunohistochemistry detected a NPM1 mutant pattern in blasts. (A) Most (>90%) areas of the bone marrow biopsy specimen are hypercellular with maturing trilineage hematopoiesis. Scattered dysplastic (small hypolobated) megakaryocytes are present. Blasts are scattered and occasionally form small clusters (A, circle). (B) At high‐power magnification, the blasts are large with irregular nuclear contours and abundant cytoplasm. Inset shows blasts on bone marrow smears, which are large with irregular nuclei, basophilic cytoplasm, and cytoplasmic blebs. (C) NPM1 immunostain shows that most cells exhibit nuclear staining only, indicating a wild‐type pattern. However, blasts that are large and scattered show both nuclear and cytoplasmic staining, indicating a mutant pattern. (D and E) In a focal area (<10%, at the edge of the core biopsy), the blasts form sheets and show diffuse cytoplasmic and nuclear NPM1 staining (F).

A 59‐year‐old man presented initially with borderline cytopenia and several subcutaneous lesions. An excisional biopsy specimen from the right leg mass obtained at another institution showed myeloid (monocytic) sarcoma. The patient came to our institution for further workup and management. A complete blood count showed anemia (hemoglobin 11.3 g/dL; normal range 13.3–17.4 g/dL), mild thrombocytopenia (135 × 10^9^/L; normal reference 160–397 × 10^9^/L), and monocytosis (58%, absolute 2.78 × 10^9^/L; normal reference 0.24–0.85 × 10^9^/L) with no circulating blasts. Bone marrow aspiration and biopsy were performed. The aspirate smears showed trilineage hematopoiesis with scattered blasts and dysmegakaryopoiesis manifested by small hypolobated megakaryocytes. The blasts were large with irregular nuclear contours, dispersed chromatin, moderate to abundant basophilic cytoplasm, and cytoplasmic blebs (Figure 1B, inset). A subset of blasts had cytoplasmic vacuoles and occasional blasts showed hemophagocytosis. The bone marrow biopsy specimen showed trilineage hematopoiesis with sequential maturation in over 90% of the medullary space (Figure 1A). Scattered dysplastic megakaryocytes were present and small clusters of blasts (Figure 1A, circled) with irregular to folded nuclei, dispersed chromatin, and abundant cytoplasm were identified. In one focus representing <10% of the total specimen (Figure 1D–F), the blasts formed sheets. IHC performed on the bone marrow biopsy specimen showed blasts with cytoplasmic and nuclear staining (Figure 1C,F) for NPM1, suggestive of *NPM1* mutation. The blasts were also positive for CD4, CD33, and CD56. In areas with sequential trilineage hematopoiesis, NPM1 immunostain showed only nuclear staining suggestive of wild‐type *NPM1* in these cells.

Flow cytometric immunophenotypic analysis performed on the aspirate specimen detected two aberrant cell populations. One population representing 0.35% of cells was CD34 positive with increased CD117 and decreased CD38 expression. The second population representing <0.1% of total cells was monocytic and positive for CD4, CD56 (bright), and CD64 with decreased CD14. The latter population corresponded to the blasts observed in the aspirate smears and biopsy specimen.

NGS of 81 gene panels initially reported mutations in *ASXL1*, *CBL*, *IDH2*, *PTPN11*, and *SRSF2* without *NPM1* (based on the calls made by the automated variant caller). The RNA translocation panel (108 genes, including *NPM1*) did not identify any fusion genes. Conventional karyotypic analysis showed 90–94,XXYY,i(1)(q10),+8,−11,+12,−18,+21[cp4]/46,XY[16]. Optical genome mapping did not detect any structural variants. Based on the mutant NPM1 staining pattern detected by IHC, the NGS results were reviewed manually and a *NPM1* canonical mutation (NM_002520, c.860_863dupTCTG, p.W288fs) with a variant allele frequency less than 2% was detected.

The diagnosis of acute myeloid (monoblastic) leukemia with *NPM1* mutation was established. The presence of monocytosis, trilineage hematopoiesis, and morphologic dysmegakaryopoiesis is suggestive of chronic myelomonocytic leukemia in the background. The patient was treated with FLAG‐IDA (fludarabine, high‐dose cytarabine, idarubicin and G‐CSF (granulocyte colony‐stimulating factor)) and venetoclax induction therapy. Bone marrow aspiration and biopsy performed at the end of cycle one showed morphologic remission. However, there was persistent monocytosis and extramedullary disease.

## 3 DISCUSSION

In most cases of AML with mutated *NPM1*, IHC assessment for NPM1 shows nuclear and cytoplasmic staining [7, 8, 13]. However, occasional discrepancies between IHC results and molecular analysis have been described in the literature, including cases with a mutant pattern suggested by IHC but negative for mutation by molecular analysis, as well as cases with a wild‐type pattern by IHC but positive for mutation by molecular analysis [13, 14]. Our case falls into the former category of discrepancy, and at least three scenarios could account for this type of disparity (aberrant pattern detected by IHC but negative by molecular analysis): (1) target sequencing may have been designed to only cover exon 12 of *NPM1*, and therefore cannot detect rare mutations in other exons [10, 11, 12]; (2) *NPM1* rearrangement may have occurred which can only be detected by fluorescence in situ hybridization (FISH), an RNA translocation panel, or optical genome mapping but not by targeted DNA sequencing [9, 10]; or (3) the blasts in AML can have a patchy or focal distribution in the bone marrow, and as a result DNA sequencing can show a false‐negative result due to a low blast count [14]. The last scenario was demonstrated in the case we report here, highlighting the importance of IHC as a screening/surrogate marker for *NPM1* mutation. In this study, the IHC result triggered a manual review of the NGS data for identification of variants with low allele frequency. In addition to the scenarios mentioned above, NPM1 IHC can be useful in cases with a “dry tap” or extramedullary tissue biopsy specimens involved by AML that might not be triaged for molecular analysis.

It is important to be aware that interpretation of NPM1 IHC requires some experience. Woolthuis et al. investigated five cases of AML in which *NPM1* mutation was detected by sequencing, but IHC showed a wild‐type staining pattern [14]. In their study, three cases were due to inter‐observer variability in the evaluation of IHC results. Reexamination of the three cases showed cytoplasmic staining in a minority of cells, small clusters of myeloblasts, or in a thinner tissue section [14]. The discrepancy in the other two cases were likely due to fixation and histotechnical factors generating a false‐negative staining result [14].

In summary, evaluation of NPM1 mutation status is critical for the diagnosis and clinical management of AML patients. We agree with others who have proposed that IHC methods should be combined with molecular techniques (polymerase chain reaction, NGS, FISH or translocation) to thoroughly assess for *NPM1* genetic alterations [14]. Despite the advances of molecular techniques in AML diagnosis, the traditional technique of IHC continues to play a role in detection of *NPM1* mutations.

## AUTHOR CONTRIBUTIONS

Wei Wang designed the research. Qing Wei, Sa A. Wang, Sanam Loghavi, Hong Fang, L. Jeffrey Medeiros, and Wei Wang wrote and approved the manuscript.

## CONFLICT OF INTEREST STATEMENT

The authors declare that there is no conflict of interest that could be perceived as prejudicing the impartiality of the research reported.

## FUNDING INFORMATION

The authors received no specific funding for this work.

## ETHICS STATEMENT

The study was performed in accordance with the principles of the Declaration of Helsinki and the institutional guidelines.

## PATIENT CONSENT STATEMENT

The authors have confirmed patient consent statement is not needed for this submission.

## PERMISSION TO REPRODUCE MATERIAL FROM OTHER SOURCES

The authors confirm that no permission was required for the article.

## CLINICAL TRIAL REGISTRATION

The authors have confirmed clinical trial registration is not needed for this submission.

## Data Availability

No datasets were generated in this study.
